# Treatment of Pertussis

**Published:** 1879-12

**Authors:** 


Treatment of Pertussis.—Dr. J. Lewis Smith, in the October number of the *American Journal of Medical Sciences*, recommends the following formula to be used as a spray, and inhaled in pertussis:

℞—Acid carbolic....................ʒss

Potas. chlorat.................... ʒij

Glycerine........................℥ij

Aquæ.. ..........................℥vi, misce

The spray to be administered three times daily, from two to five minutes at each sitting.

Dr. Smith says the good effect of the spray seems to be largely due to the carbolic acid, which, when used locally, is known to produce an anæsthetic effect on mucous surfaces, but in one or two instances in which the chlorate was temporarily omitted from the mixture, patients seemed to do better with than without it.

